# Response to Kantor’s “The great automatic grammatizator: On the use and misuse of large language models in scientific and academic writing”

**DOI:** 10.1016/j.jdin.2025.02.005

**Published:** 2025-04-09

**Authors:** Dag Øivind Madsen, Shahab Saquib Sohail

**Affiliations:** a USN School of Business, University of South-Eastern Norway, Hønefoss, Norway; b School of Computing Science and Engineering, VIT Bhopal University, Bhopal, Madhya Pradesh, India

**Keywords:** academic writing, artificial intelligence, equitable access, large language models, research, scientific writing

*To the Editor:* We commend^1^ Dr Kantor for providing a thought-provoking perspective on the growing impact of large language models (LLMs) like ChatGPT, Claude, and Google Gemini on scientific and academic writing. The letter presents a balanced view of LLMs’ potential to democratize access to high-quality language support, especially for non-native English speakers, while also cautioning against overreliance. However, we believe the discussion could benefit from further nuance regarding equity, authorship, and the evolving role of human researchers in an AI-enhanced academic environment.

Firstly, the letter highlights how LLMs can equalize academic writing. Kantor highlights the advantages of researchers with deep pockets. Research has shown great inequality among colleges and universities within and across countries.^2^ For example, historically, researchers in resource-constrained settings (especially in the Global South) have faced barriers due to limited access to costly professional editorial support and proofreading services. LLMs offer a cost-effective alternative, potentially leveling the playing field. However, this benefit is still not completely universal. The quality of LLM outputs is inherently tied to internet access and computational resources, which remain unevenly distributed globally.^3^ Additionally, there is a growing trend toward tiering of chatbot services, such as ChatGPT's new $200-a-month Pro subscription, which relatively few academics can afford. Addressing these disparities is essential if LLMs are to fulfill their promise of equitable access. The growing open-source LLM ecosystem could possibly be a solution.

Kantor also discusses concerns about the opaque nature of LLM outputs, likened to an “invisible brainˮ. This concept is akin to the “black boxˮ analogy often used in AI discourse, highlighting the lack of transparency in how inputs are processed into outputs.^4^ It further highlights the importance of transparency in AI design. Developers of AI tools should prioritize features that provide explainability, such as tracing outputs back to training data or offering confidence scores. Such measures can help alleviate discomfort and foster trust in AI tools.

Furthermore, the letter mentions the potential use of LLMs in tasks like simple qualitative data analysis and coding. While some authors suggest that LLMs can be applied to qualitative researchers,^5^ it remains uncertain whether they can reliably replicate these qualitative methods at their current level of sophistication. While LLMs are evolving rapidly, their application in qualitative research raises significant methodological and ethical concerns that warrant caution. Coding and thematic analyses require nuanced understanding and contextual judgment, and human oversight is indispensable to ensure validity.

We agree with Kantor’s view that flexibility and openness are necessary during this transitional period. The academic community must cultivate strategies that enable researchers, students, and other writers to engage with LLMs critically. By doing so, we can ensure that these tools complement, rather than replace, human ingenuity and judgment.

Sincerely,

## Declaration of generative AI and AI-assisted technologies in the writing process

While preparing this work, the authors used ChatGPT-4o to edit and enhance the manuscript’s language and style. After using this tool, the authors reviewed and edited the content as needed. They take full responsibility for the manuscript’s content.

## Conflicts of interest

None disclosed.
